# Clustered DNA Damage: Electronic Properties and Their Influence on Charge Transfer. 7,8-Dihydro-8-Oxo-2′-Deoxyguaosine Versus 5′,8-Cyclo-2′-Deoxyadenosines: A Theoretical Approach

**DOI:** 10.3390/cells9020424

**Published:** 2020-02-12

**Authors:** Boleslaw T. Karwowski

**Affiliations:** DNA Damage Laboratory of Food Science Department, Faculty of Pharmacy, Medical University of Lodz, ul. Muszynskiego 1, 90-151 Lodz, Poland; Boleslaw.Karwowski@umed.lodz.pl

**Keywords:** clustered DNA damage, 5′,8-cyclo-2′-deoxyadenosines, charge transfer, glycosylases, base excision repair, DFT

## Abstract

Approximately 3 × 10^17^ DNA damage events take place per hour in the human body. Within clustered DNA lesions, they pose a serious problem for repair proteins, especially for iron–sulfur glycosylases (MutyH), which can recognize them by the electron-transfer process. It has been found that the presence of both 5′,8-cyclo-2′-deoxyadenosine (cdA) diastereomers in the ds-DNA structure, as part of a clustered lesion, can influence vertical radical cation distribution within the proximal part of the double helix, i.e., d[~oxoGcAoxoG~] (7,8-dihydro-8-oxo-2′-deoxyguaosine - ^oxo^dG). Here, the influence of cdA, “the simplest tandem lesion”, on the charge transfer through *ds*-DNA was taken into theoretical consideration at the M062x/6-31+G** level of theory in the aqueous phase. It was shown that the presence of (5′*S*)- or (5′*R*)-cdA leads to a slowdown in the hole transfer by one order of magnitude between the neighboring dG→^oxo^dG in comparison to “native” *ds*-DNA. Therefore, it can be concluded that such clustered lesions can lead to defective damage recognition with a subsequent slowing down of the DNA repair process, giving rise to an increase in mutations. As a result, the unrepaired, ^oxo^dG: dA base pair prior to genetic information replication can finally result in GC → TA or AT→CG transversion. This type of mutation is commonly observed in human cancer cells. Moreover, because local multiple damage sites (LMSD) are effectively produced as a result of ionization factors, the presented data in this article might be useful in developing a new scheme of radiotherapy treatment against the background of DNA repair efficiency.

## 1. Introduction

Each human body cell is exposed to ionization radiation (IR), environmental factors, metabolic processes, etc*.,* which can lead to the formations of DNA lesions. The interaction of the above factors with the genome gives rise to ~10^5^ damage events per day per cell. Due to the mutual DNA damage position, the isolated and clustered (CD) types of lesion are specified [1]. A CD is defined as two or more lesions formed in a single helix turn [2]. IR can produce DNA damage directly by direct energy deposit and indirectly by reactive oxygen species (ROS) generation, e.g., water ionization. Of all ROS, the hydroxyl radical (^●^OH) is the most reactive. Each GC and AT base pair in *ds*-oligo is hydrated by 44 and 27 H_2_O molecules, which form the first solvation layer [3]. As a result, due to the ^●^OH lifetime of ~8.7 × 10^−9^ s and diffusion distance of 9.3 nm, which is three times larger than a double helix turn, it can react with sugar and the base moieties of oligonucleotide and cause different types of DNA damage [4]. The most preferred interaction of hydroxyl radical with (a) nucleobases, is its addition reaction to double bonds and (b) hydrogen atom abstraction from a 2-deoxyrybose moiety of nucleotides. However, the following order of hydrogen abstraction from the sugar–phosphate backbone has been found: H5′>H4′>H3′~H2′~H1′ [5]. This observation is, therefore, well connected to their interaction order with the first ds-DNA solvation layer [6]. Moreover, the experimental work of Chatgilialoglu et al. on the reaction of hydroxyl radicals with 2′-deoxypurines has shown that 25% of hydrogen atom abstraction occurs on the 2-deoxyribose, 40% of which leads to C5′ radical formation [7]. The lesion events induced by IR and by normal aerobic metabolism are structurally similar. The modification formed independently of the source can threaten the stability and reproducibility of genomic information. Fortunately, most DNA lesions formed in cells are removed from the genome by various repairs. Base excision repair (BER) can be regarded as a front-line defense that effectively removes a single/isolated DNA lesion [8]. Additionally, DNA damage formation in early mitotic cells can lead to mitosis reversal [9]. The CDs are removed much more slowly from the genome, depending on the damage type and its amount [10]. It has been found during the CD repair process that a hierarchy of their subunit/component damage removal/repair exists, e.g., the apurinic/apirimidinic site (AP) as well as single strand brake (SSB) have a negative influence on the vicinal nucleic base lesion excision that is not removed before AP site or SSB repair [11]. This cell strategy limits the formation of double-strand breaks (DSBs). However, DSBs that are not repaired properly can lead to a loss of heterozygosity or chromosomal rearrangements for diploid organisms and in the case of a haploid is frequently lethal [12]. Moreover, during local multi-damage site (LMDS) repair, the probability of mutagenesis increases [10]. As a result, the low fidelity of repair machineries or high lesion resistance (like (5′*R*)/(5′*S*)cdA) can lead to many different disease processes, cancer, aging, neurodegenerative disease, etc. [13]. Of the many reviews dedicated to clustered DNA lesions published thus far, that of Georgakilas et al. would seem to be the most valuable to the scientific audience [14]. From a medical point of view, radiotherapy solely or radiotherapy coupled with chemotherapy is one of the most effective anticancer treatments [15]. Due to the nature of radiation type two, Low- and High linear energy transfer (LET) should be taken into consideration in the context of DNA lesion formation [16]. It is important to mention here that anticancer boron neutron capture therapy (BNCT) is very attractive as a therapeutic treatment from the point of well-targeted radiotherapy with a simultaneous reduction in side effects [17,18]. In this medicinal course of treatment, the boron carrier compound, containing ^10^B isotopes, after parenteral administration, reaches a higher concentration in the tumor cells than in healthy tissue [19]. Due to the nature of ^10^B, the “pro-anticancer drug” is not radioactive, and the biostability of the boron carrier, e.g., L-boronophenylalanine, means it is nontoxic. The situation is different when the changed tissue is radiated by low (thermal) energy neutrons that thermalize in tissue and interact with the ^10^B nuclei. As a result, high linear energy transfer (LET) alpha and lithium particles are produced, destroying the surrounding cells. Additionally, High-LET results in the propagation of the reactive oxygen species (ROS), which can force different types of DNA lesion formation. Therefore, DNA damage formations inside a tumor cell genome have been noticed as the core of radiotherapy activity constructed on both Low- and High-LET radiation [20]. As shown, the number and complexity of LMDS are proportional to the radiation dose (ionization density). Around 90% of observed DSBs have their source in other DNA damage generated by IR [21,22]. Modified bases, such as ^oxo^dG, at the beginning of BER processes, are directly converted to the AP site or single-strand break by specific glycosylates, either mono- or bi-functional. These proteins, either solely or coupled with endonucleases, at the beginning of BER process, can convert the non-DSB clusters to the double-strand break′s side [8]. Due to its high genotoxicity (i.e., DSB), the cell tries to avoid their production [23,24]. To elucidate these phenomena, numerical simulations have been used to visualize and investigate the influence of a DNA lesion on its spatial geometry as well as to demonstrate oligonucleotide interaction with different types of repair enzymes at an atomistic level [24,25,26]. The genome of a human cell contained 3x10^9^ bases in comparison to *Escherichia coli* (*E. coli*)*,* which contained only ~5 × 10^5^ [27,28].

**Figure 1:**
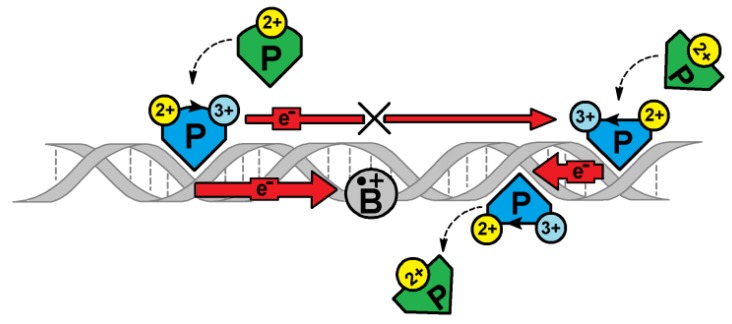
A schematic overview of [4Fe-4S] glycosylase (P - MutyH) DNA damage (B - ^oxo^dG) recognition under *ds*-DNA charge transfer mode (red arrow). Glycosylase in its second oxidation state [4Fe-4S]^2+^ binds to *ds*-DNA and subsequently converts to [4Fe-4S]^3+^. If a released e^-^ migrates, without obstacles, through the double helix, reduction may result, and the release of a second protein attached to the oligo. The DNA damage cation radical appearing on the CT path causes its end by B^●+^ reduction. At this stage, P starts to migrate to the B location and recognizes and removes it. The oxidized protein binding to the *ds*-DNA is ~1000 higher than for its [4Fe-4S]^2+^ form [33].

This life code is guarded by permanently “active” repair proteins that continuously look for DNA damage. In the classical model, glycosylases tested the hydrogen bond strengths of complementary bases during their journey through the double helix rails [29]. Even though the above strategy is meticulously precise, the time needed for the whole genome to be scanned is too long to be effective for only 30 copies of MutY and 500 of EndoIII glycosylases in an *E. coli* cell [30]. Recently, Barton et al. proposed a mechanism of DNA lesion searching based on charge transfer [20,31]. It is well established that a radical cation within the double helix can migrate over 200 Ǻ away from its formation site in both directions [32]. Based on this, proteins, such as MutyH and MutY, can effectively search for a DNA damaged site after binding to the genome (Figure 1).

**Figure 2:**
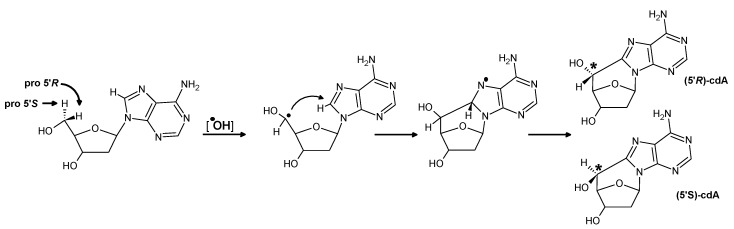
The possible reaction path of (5’*R*) and (5’*S*) 5’,8-cyclo-2’-deoxyadenosine formation [47].

The charge migration (CT) toward π-stacked base pairs (BP) is sensitive to its structural and electronic changes. Voityuk [34,35] has shown that the hole migration process between stacked base pairs in ds-DNA can take place between triplet excited states [36,37,38]. From this point, the 8,7-dihydro-8-oxo-2′-deoxyguanosie, which shows a lower ionization energy/potential (6.94 eV [22,39]) than its precursor dG, can easily be oxidized and constitutes an endpoint for cation radical migration, which leads to a) protection of the rest of *ds*-oligo against oxidation, and b) flagging the lesion for glycosylase movements. Of over 80 different types of DNA damage, ^oxo^dG is the most common (600+/− 170 per 10^6^nucleosides) [40]. Its level of formation depends on the genome′s exposure to harmful factors. Consequently, it is a good marker for carcinogenesis, aging, as well as radiotherapy treatment. At the other end of frequency scale lies (5′*R*)/(5′*S*)-cdA, for which the level was noted as 0.07/0.93 × 10^6^ nucleosides [40]. However, the frequency of cdA depends on the source/tissue taken into consideration, as well as the techniques used for detection [41,42]. The cdA is the product of C5’-C8 cyclization initiated by one of the C5’ proton ^●^OH abstractions [43]. It should be pointed out here that cdA is the simplest tandem lesion. By definition, clustered DNA damage consisting of two continuously damaged nucleosides is referred to as tandem lesions [44]. The pioneering work of Box suggested the possible formation of tandem lesions consisted of two adjacent modifications, e.g., sugar and base moieties resulting from a single free radical initiating event [45,46]. The mechanism of 5′,8-cyclo-2′-deoxypurine formation clearly shows that cdA (tandem lesion) is the single nucleoside in which the base and sugar moieties have been modified by a single hydroxyl radical event (Figure 2) [47]. The experimental data shows that the (5′*R*)-cdA is formed predominantly (5′*S*)-cdA in the cases of free nucleoside or single-stranded DNA, while both diastereomers are produced in similar amounts in double-stranded DNA [48].

Both diastereomers of 5’,8-cyclo-2’-deoxyadenosine are a substrate for the nucleotide excision repair (NER) system; until now, unspecified glycosylases were known for removing them. (5’*R*)- and (5’*S*)-cdA are 40 and 150 times more slowly ejected from the genome than *cis*-platin adducts [49]. This indicates that their appearance in the double helix structure constitutes a serious problem even for NER machinery. Moreover, the long lifetime of cdAs in the genome predispose it to be a part of a clustered lesion, especially when tissue has been exposed to IR. Conversely, to 5’,8-cyclo-2’-deoxyadenosine, a ^oxo^dG lesion is easily repaired by BER. It should be mentioned that ^oxo^dG is formed by a different dG reaction, e.g., with ^1^O_2_ or by dG^●+^ with H_2_O [50]. Saito et al. have shown that dG and dG clusters (GG, GGG, GGGG) within *ds*-DNA constitute the sink for the hole migration, due to their ionization potentials (IPs) being lower than other nucleic bases [51]. The G on the 5’ site of the bunch is the more easily ionized. Moreover, the IP of G depends strongly on the base attached to its 3’-end but only negligibly to that on the opposite 5’ OH group [52].

## 2. Materials and Methods

### 2.1. Computation Methodology of QM/MM (Quantum Mechanics/Molecular Mechanics) Studies

**Figure 3:**
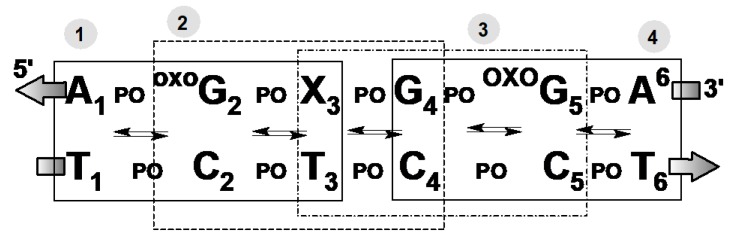
Graphical representation of *ds*-oligonucleotides divided into four trimes (indicated by dashed squares): ^oxo^dG_2,4_DNA (X = dA); RcdA^oxo^dG_2,4_DNA; (X = (5’*R*)-cdA); ScdA^oxo^dG_2,4_DNA (X = (5’*S*)-cdA).

The geometry optimizations of ds-hexamers (Figure 3), i.e., ^oxo^dG_2,4_DNA, RcdA^oxo^dG_2,4_DNA, and ScdA^oxo^dG_2,4_DNA, were performed using QM/MM strategy [53,54]. The structures of the mentioned systems were divided into high-HL (nucleobases, M06-2X/D95*) and low-LL (sugar-phosphate backbone, UFF (universal force field)) levels (layers) of calculation using the ONIOM (our own n-layered integrated molecular orbital and molecular mechanics) method [55,56]. Due to the complexity of the system, the negative charges of all the phosphate groups were neutralized by the addition of protons. This strategy has been well documented as applicable to charge/proton transfer for structural studies of nucleic acids [57,58] as well as being adopted for low electron migration between base aromatic rings and a sugar–phosphate backbone [59].

The optimized nucleotide complexes (ds-hexamers) were converted to nucleobase pairs, which were used for inter- and intrastrand interaction energy calculations. The sugar–phosphate backbone was removed from the obtained structures, leaving suitable base pair systems with subsequent atom saturation with the necessary hydrogen atoms. The spatial location of the hydrogen atoms added for saturation was optimized at the M06-2X/D95* level of theory in the aqueous phase, with the position of all other atoms frozen. 

### 2.2. Computation Methodology of DFT Study

All energy calculations were performed in the condensed (aqueous) phase by density functional theory (DFT) using the M06-2X functional with augmented polarized valence double-ζ basis set 6-31+G** [60,61]. The characterization of the transition dipole moment of excited states and the single point calculation at the M06-2X/6-31+G** level of theory were performed using time-dependent DFT (TD-DFT) methodology [62]. The solvent effect was described for an aqueous medium, applying Tomasi’s polarized continuum model (PCM) [11,63]. For all optimized structures, a charge and spin analysis was achieved using the Hirshfeld methodology at the M06-2X/6-31+G** level [64]. The electron coupling was calculated using the Generalized Mulliken–Hush methodology [65]. The electronic properties, i.e., adiabatic ionization potential (AIP), vertical ionization potential (VIP), vertical electron attachment energy (VEAE), and nuclear relaxation energies (NER1 and NER2) were calculated as previously described [66]. The energy (*E*_geometry_^charge^) of the molecule (neutral form) is described as *E*_0_^0^, the vertical cation as *E*_0_^+^, the adiabatic cation as *E*_+_^+^, and the vertical neutral as *E*_+_^0^.

All calculations were performed in the aqueous phase on Gaussian 09 (revision A.02) software package [67].

The three-dimensional structural analyses of the mentioned ss- and ds-DNAs, based on a standard reference frame, were obtained by a 3DNA software package using the web-based interface w3DNA (web 3DNA) [68].

## 3. Results and Discussion

Bearing in mind the above, in these studies, the influence of (5’*R*)- and (5’*S*)-cdA on (a) vicinal ^oxo^dG ionization potential, as well as on (b) the radical cation (hole) transfer within the double helix were taken into consideration. To this end, three ds-oligos were chosen in which cdA is surrounded by ^oxo^dGs to form a single-stranded clustered damage site, as shown in Figure 3. This choice is partially supported by the previous observation that (5’*S*)-cdA impacts on the BER efficiency of dU repair in a clustered damage context [69]. In the experimental studies mentioned, it was shown that the appearance of cdA in the structure of a single or double-stranded oligonucleotide can stop the initial base repair enzymes activation, i.e., uracil-DNA glycosylase (UDG) and human AP-site endonuclease 1 (HAPE1) depending on the cdA diastereomeric forms. However, nothing is known about the influence of cdA on the DNA lesion recognition process by glycosylases in the context of clustered damage.

The initial spatial structures of complete ^oxo^dG_2,4_DNA, RcdA^oxo^dG_2,4_DNA, and ScdA^oxo^dG_2,4_DNA were optimized using the ONIOM strategy at the M062x/D95*: UFF level of theory in a condensed phase [70,71]. The RMSD (Root Mean Square Deviation) calculation for the suitable neutral and radical cationic forms of ds-oligonucleotides elucidated that the loss of an electron by ScdA^oxo^dG_2,4_DNA leads to reduced geometry distortion (RMSD = 0.226Å), while for ^oxo^dG_2,4_DNA and RcdA^oxo^dG_2,4_DNA, the distortion was found to be significantly higher, i.e., 1.337Å and 1.194Å, respectively. This indicates that the presence of (5’*S*)-cdA renders the 3D structure of the double helix almost radical cation resistant.

The electronic parameters discussed below were calculated at the M062x/6-31+G** level of theory in an aqueous phase for the systems in which the sugar–phosphate backbone was removed and replaced by hydrogen atoms in suitable nucleic base positions (N1, N9, or C8). An analysis of the stacking energy interaction (*E*_ST_) and BP ring overlap surface (*S*_BP_) between neighboring base pairs, and the differences between the neutral and radical cation forms of the investigated *ds*-oligo, confirms the RMSD observation. The absolute average values |*ΔE*_ST_| and |*ΔS*_BP_| were found as follows (in kcal/Å^2^): ^oxo^dG_2,4_DNA 1.22/3.05; ScdA^oxo^dG_2,4_DNA 0.62/0.43; RcdA^oxo^dG_2,4_DNA 1.42/1.11, respectively. Following the work of Rothlisberg [72] and Senthilkumar [52], the *ds*-oligonucleotides presented in Figure 3 were divided into four trimes as denoted (the notification has been simplified to the base sequence of the purine strand). Contrary to previous results, here the spatial geometry of each *ds*-oligo form was fully optimized. The charge transfer through the double helix can be perceived as an iterative superexchange or multistep hopping process [73]. 

**Figure 4:**
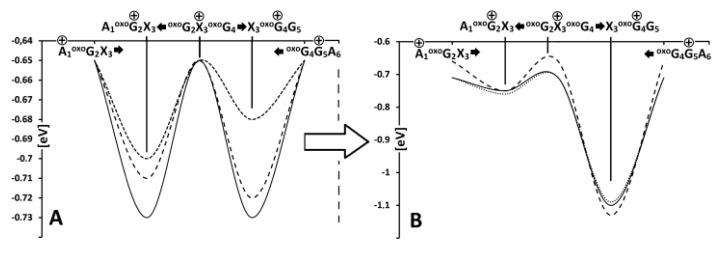
The energy barrier profile of the charge migration process through the double helix containing clustered lesions in vertical (**A**) and adiabatic (**B**) modes, as follows: --- A_1_^oxo^G_2_A_3_^oxo^G_4_G_5_A_6_; ••• A_1_^oxo^G_2_(5’*R*)-cdA_3_^oxo^G_4_G_5_A_6_; ^___^ A_1_^oxo^G_2_(5’*S*)-cdA_3_^oxo^G_4_G_5_A_6_.

Therefore, the following energy barriers of radical cation transfer can be assigned A_1_^+oxo^G_2_X_3_→ A_1_^oxo^G_2_^+^X_3,_ X_3_^+oxo^G_4_G_5_ → X_3_^oxo^G^+^_4_G_5_, and ^oxo^G_4_G^+^_5_A_6_ → X_3_^oxo^G^+^_4_G_5_ for all investigated hexamers, where X = dA or (5’*S*)-cdA or (5’*R*)-cdA (Figure 3). All the calculated energies are presented in the Supplementary Materials (Tables S1 and S2). Following the nature of the charge migration process, the energies of the donor and acceptor were described as the sum of the energies of suitable base pairs, extracted from an adequate hexamer, for example, a) vertical mode: the energy of donor A_1_*(E_+_^+^),* ^oxo^G_2_*(E_0_^0^),* X_3_*(E_0_^0^)* and acceptor A_1_*(E_0_^0^),* ^oxo^G_2_*(E_+_^0^),* X_3_*(E_0_^+^*), and b) adiabatic mode: the energy of donor A_1_*(E_+_^+^),* ^oxo^G_2_*(E_0_^0^),* X_3_*(E_0_^0^)* and acceptor A_1_*(E_0_^0^),* ^oxo^G_2_*(E_0_^0^),* X_3_*(E_+_^+^*). The energy “barrier” (base pair system) is described as the sum of A_1_(*E_+_^0^*)*,* ^oxo^G*_2_*(*E_0_^+^*)*,* X_3_(*E_0_^0^*) energies in the vertical or A_1_*(E_0_^0^),* ^oxo^G_2_(*E_+_^+^*)*,* X_3_(*E_0_^0^*) in the adiabatic mode. Using the above-described strategy, the barrier (Δ*G*) for hole transfer within interlaced trimers were assigned in vertical and adiabatic modes (Figure 4) [74]. For the vertical mode, it was found that the radical cation appearing on adenine (A_3_) surrounded by two 7,8-dihydro-8-oxo-guanosines (^oxo^G_2_ and ^oxo^G_4_) was able to preferably migrate to ^oxo^G_4_ (*ΔG*_A3__→_^oxo^_G2_=-0.71eV; *ΔG*_A3__→_^oxo^_G4_ = −0.72eV). Replacing 2’-deoxyadenine (A_3_) in the structure of the investigated native hexamers by one of the cdA diastereomers had different results: the *ΔG* found for (5’*R*)-cdA indicated a hole transfer toward ^oxo^G_2_ ((*ΔG*_(5’*R*)-cA3__→_^oxo^_G2_ = −0.70eV; *ΔG*_(5’*R*)-cA3__→_^oxo^_G4_ = −0.68eV), but surprisingly no difference in *ΔGs* (−0.73eV) was observed for trimers possessing (5’*S*)-cdA. The charge rearrangement time in *ds*-oligo was estimated as 5 × 10^3^ s^−1^ [73]. Following the relaxation of the *ds*-oligos structure, the results obtained for*ΔGs* in the adiabatic mode elucidated for all cases independently of the nucleo-base at position 3 (A or cdA) a preference for radical cation migration to ^oxo^G_4_.

The charge transfer migration through the double helix can be described according to the Marcus theory, which states that the rate constant (*k*_ET_) of CT depends on several factors: the structure of π-stacks, i.e., BPs, the driving force (*ΔF*), nuclear reorganization (λ), activation (*E*_a_), and the electron-coupling (*V*_da_) energies [75]. It is important to mention that, in this study, the triplet excitation energy of all investigated BP dimers (stacks) was found to be the lowest (Table S2). The *V*_da_ was calculated according to the GMH (generalized Mulliken–Hush) strategy within the terms of the occupied Kohn–Sham orbital method [76,77]. The charge transfer, which passes through the adiabatic states of donor and acceptor, is associated with the movement of internal geometries (atoms), expressed by λ in the Marcus theory. An analysis of the reorganization energies revealed a significant rise for the ^oxo^G_2_X_3_^oxo^G_4_ (X = A or (5’*R*)/(5’*S*)-cdA) systems (Table 1, Table S3). It indicated that these parts of *ds*-oligo play a significant role in CT as a radical cation point of destiny. Due to the fact that *k*_ET_ is strongly dependent on the distance between donor and acceptor, an influence of the cluster DNA lesion on charge transfer in the double helix shape can be expected.

A comparison of the calculated *K_HT_* value between the base pair dimers of the discussed *ds*-oligo showed that the presence of (5’*R*)- and (5’*S*)-cdA slowed down G_5_→^oxo^G_4_ radical cation migration by one order of magnitude more than was observed for ^oxo^dG_2_,_4_DNA. The differences of influence between both cdA diastereomers on hole transfer were well visible for X_3_→^oxo^G_4_ (X_3_ = A or (5’*R*)-, (5’*S*)-cdA). As Table 1 and Table S4 shows, the presence of (5’*R*)-cdA within *ds*-oligo leads to *K_HT_* increases by one order of magnitude in comparison with *S*cdA^oxo^dG_2,4_DNA and ^oxo^dG_2,4_DNA. These observations indicate that cdAs appearing as part of clustered damage can lead to different biochemical and thermodynamical results depending on the C5’ chirality. For the remaining base pair dimers of DNAs with LMSD, the *K_HT_*, as well as λ, adopts a value around zero, which suggests that this part of the *ds*-oligo only plays a transfer role during the CT process. These results are further in good agreement with the vertical (VIP) and adiabatic (AIP) ionization potentials of each base pair calculated at the same level of theory. For this purpose, BPs were isolated from suitable forms of *ds*-oligos and noticed to be either stable radical cations or vertical ones. The lowest AIPs (5.53–5.57eV) were found for ^oxo^G_4_C_4_ in cases of all the *ds*-oligos investigated. For the second pair with 7,8-dihydro-8-oxo-guanosine, the values were higher by ~0.3eV. Due to the nature of the double helix, the PCM (polarizable continuum model) was used in this study instead of NEPCM (nonequilibrium PCM) for vertical ionization potential calculations [78]. The reason is two-fold: firstly, the NEPCM was validated for single nucleosides and nucleotides that are far from the base pair dimer (stacks); secondly, the double helix interacts with the first solvation layer only by its outer shape. Therefore, for the sake of cohesion in this study, which is spread from base pair to ds-hexamer, the classical PCM model was used.

**Figure 5:**
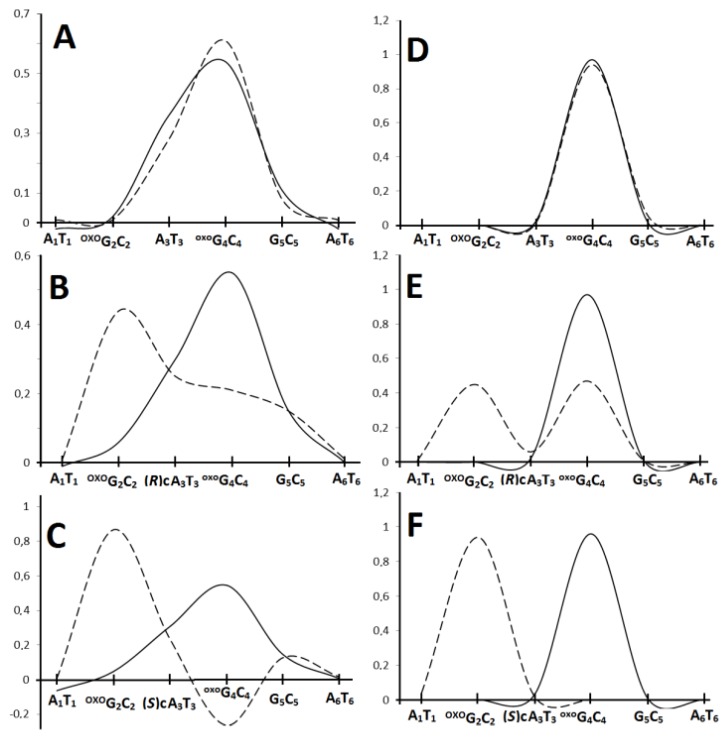
Vertical (---) as well as adiabatic (^___^) Hishfeld charge and spin density distribution in a purine strand of the investigated ds-oligonucleotides; the base pair notation has been used for clarity, (**A**–**C**) and (**D**–**F**), respectively. ^oxo^dG_2,4_DNA (**A**,**D**); ScdA^oxo^dG_2,4_DNA (**B**,**E**); RcdA^oxo^dG_2,4_DNA (**C**,**F**). The whole row data is presented in Table S5.

In the vertical cation mode/state, the lowest calculated VIP (eV) value of base pairs was found as follows: ^oxo^G_2_C_2_ (5.94) of RcdA^oxo^dG_2,4_DNA, ^oxo^G_4_C_4_ = ^oxo^G_2_C_2_ (5.93) of ScdA^oxo^dG_2,4_DNA, and ^oxo^G_4_C_4_ (5.95) of ^oxo^dG_2,4_DNA. Table S6 presents each base pair adiabatic and vertical ionization potential extracted from *ds*-oligonucleotides: ^oxo^G_2,4_DNA, RcdA^oxo^G_2,4_DNA, ScdA^oxo^G_2,4_DNA, calculated at the M062x/6-31+G** level of theory in the aqueous phase. The negligible differences between VIP and AIP of the remaining base pairs well support the previous observation noted for λ and *K_HT_*. The negligible value of relaxation energy indicates that the spatial molecule structure is resistant to charge adoption. The AIP of (5’*R*)-cdA_3_T_3_ base pairs was assigned as higher by 0.04eV than that noted for VIP. The above results indicate that the electronic properties of *ds*-oligo change during spatial structure accommodation (adiabatic *ds*-oligo mode) to an electron loss event. Moreover, the difference between the discussed oligonucleotides with the lowest VIP field suggests that both diastereomers of cdA can affect the CT and lead to DNA damage recognition by [4Fe-4S] glycosylase [29] in the context of clustered lesions. The above electronic properties are inextricably linked to spin and charge distribution. The electron loss by *ds*-oligo leads to a suitable vertical cation formation with subsequent adiabatic form adoption. Depending on the spatial geometry of the double helix, the positive charge can migrate along the strands, in both directions, exploiting the nature of the stacked bases. For the clustered DNA lesion, which contained exclusively ^oxo^Gs, in vertical and adiabatic modes, the spin and charge mainly settled on the purine moiety of the ^oxo^G_4_C_2_ base pair, as expected (Figure 5, Table S5).

**Figure 6:**
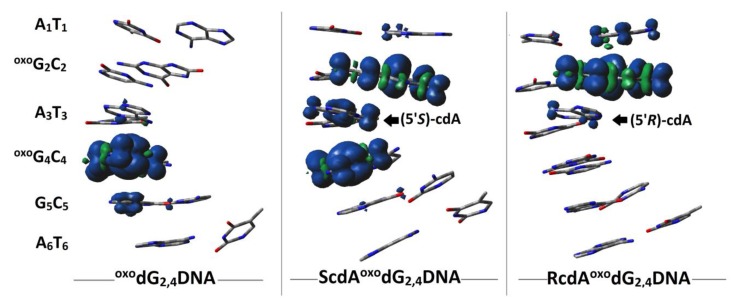
Graphical representation of spin density distribution for the vertical radical cation form of *ds*-oligos.

The ^oxo^Gs separation by one of the cdA diastereomers leads to a different distribution pattern in the initial state. The appearance of (5’*S*)-cdA forces the charge and spin density distribution over all the components of LMDS, while (5’*R*)-cdA accumulates them, opposite to ^oxo^dG_2,4_DNA, exclusively at the ^oxo^G_2_C_2_ location. The subsequent *ds*-oligo structure relaxation led to the charge and spin relocation toward ^oxo^G_4_C_4_ base pairs, as shown in Figure 6 (Table S5). It has been shown that the cell lifetime of a clustered DNA lesion was significantly lengthened compared to that of an isolated lesion [2]. Moreover, the yields of mutation during a genome repair process increase and depend on the LMSD components [79]. Based on the results presented above, it can be postulated that in the vertical mode, DNA damage recognition by 4Fe-4S glycosylase can be affected. This is due to the fact that a radical cation was located in a different position within the DNA clustered lesion (Figure 6). Glycosylates were able to migrate from both sides of the clustered lesion due to the radical cation distribution over the ^oxo^G(5’*S*)-cdA^oxo^G double helix part, as shown in Figure 6. In these cases, the electrons ejected by MutYs located on the 5′-end and 3′-end of clustered damage site, for example, can be trapped by both ^oxo^Gs radical cations. From the side of lesion detection mechanism, two glycosylases are able, almost at the same time, to recognize both ^oxo^Gs and try to reach them from both helix ends. This can result in a protein collision, due to the spatial closeness of lesions (^oxo^G_2_ and ^oxo^G_4_), which leaves them unrepaired for a long time. Furthermore, until [4Fe-4S]^3+^ clusters are in an oxidized state, glycosylases cannot dissociate themselves from the double helix to another part of the genome. The (5’*R*)-cdA appearing between the ^oxo^Gs localizes the radical cation on ^oxo^G_2_ instead of ^oxo^G_4_, as was found for ^oxo^G_2,4_-DNA (Figure 6). This can be confusing for glycosylase activity due to the presence of the second ^oxo^G in the preferred radical cation location, i.e., ^oxo^G_4_G_5_. As has been noted, further double helix relaxation gives rise to, in all the discussed molecules, a stable radical cation settled on the ^oxo^G_4_ position. In the adiabatic state, no differences in the DNA damage recognition process should be observed for ^oxo^dG_2,4_DNA, RcdA^oxo^dG_2,4_DNA, and ScdA^oxo^dG_2,4_DNA. However, based on previous studies [69], by analogy, it can be expected that ^oxo^G appearing on the 5’-end site of (5’*S*)- or (5’*R*)-cdA means the discussed clustered lesion is undigested, leading to the BER machinery stopping before it can properly begin. Therefore, the efficiency and quality of the impact of clustered DNA damage formation during radiotherapy should be taken into consideration in the light of its influence on DNA repair processes.

## 4. Conclusions

The appearance of one of the cdA diastereomers in a clustered DNA damage structure as part of the ^oxo^G_2_X_3_^oxo^G_4_ (X_3_ = (5’*R*)-cdA or (5’*S*)-cdA) sequence gives rise to various significant consequences of charge transfer. Firstly, a change in the energy barrier for radical cation migration, i.e., (5’*R*)-cdA, elucidates a preferential transfer toward 7,8-dihydro-8-oxo-2’-deoxyguanosie located on its 5’-end, while for (5’*S*)-cdA, no references were noted for damage positioned on the 5’-end and 3’-end. Subsequently, for ^oxo^Gs separated by dA, the lowest barrier was found for hole hopping toward the 3’-end ^oxo^G. Secondly, for the CT in the vertical mode, the lowest vertical ionization potential was found as follows: for ^oxo^G_2_ of RcdA^oxo^G_2,4_DNA, ^oxo^G_2_/^oxo^G_4_ of ScdA^oxo^G_2,4_DNA, and ^oxo^G_4_ of ^oxo^G_2,4_DNA. No differences were observed in the adiabatic radical cation *ds*-oligos state, i.e., ^oxo^G_4_ always adopted the lowest AIP value. Thirdly, the presence of (5’*S*)- or (5’*R*)-cdA in LMDS leads to a slowdown of the hole transfer by one order of magnitude between the neighboring dG_5_→^oxo^dG_4_ in comparison to “native” *ds*-DNA. Therefore, this type of clustered lesion can cause defective damage recognition by [4Fe-4S] glycosylases and a reversal of the DNA repair process with the subsequent possibility of mutation production. Moreover, as local multiple damage sites are effectively produced by ionization factors, the presented results can be useful for a new scheme of radiotherapy treatment in the context of DNA repair efficiency.

## Figures and Tables

**Table 1:** The λ[eV] and *K*_HT_ [s^−1^] of hole transfer between base pairs, calculated at the m062x/6-31+G** level of theory in the aqueous phase.

HT System	^oxo^dG_2.4_DNA		*S*cdA^oxo^dG_2.4_DNA		*R*cdA^oxo^dG_2.4_DNA	
	X = dA		X = (5′*S*)cdA		X = (5′*R*)cdA	
	λ	*K_HT_*	λ	*K_HT_*	λ	*K_HT_*
A_1_→^oxo^G_2_	0.04	0.00	0.02	0.00	0.04	0.00
^oxo^G_2_←X_3_	0.03	0.00	0.01	0.00	0.05	0.00
X_3_→^oxo^G_4_	0.41	9.8 × 10^9^	0.38	5.65 × 10^9^	0.41	2.53 × 10^10^
^oxo^G_4_←G_5_	0.38	2.1 × 10^14^	0.37	6.95 × 10^13^	0.34	3.16 × 10^13^
G_5_←A_6_	0.01	0.00	0.00	0.00	0.00	0.00

## Supplementary Materials

The following are available online at https://www.mdpi.com/2073-4409/9/2/424/s1, Figure S1: Graphical representation of electron movement during the hole transfer process of the investigated *ds*-oligonucleotides, Table S1: The energy barriers (in eV) for radical cation migration between base pairs within trimers. Vertical mode, i.e., the energies of each base pair’s radical cation, which were calculated for their neutral geometry. Adiabatic mode i.e., the energies of each base pair’s radical cation were calculated for their cation geometry. Arrows indicate direction of Hole Transfer from one base pair to another e.g., A^+^ → G calculate at the M062X/D95*//M062x/6-31+G** level of theory in the aqueous phase, Table S2. The energies (in Hartree) of Neural, Vertical Cation, Adiabatic Cation and Vertical Neutral forms of base pairs extracted from *ds*-oligonucleotides calculated at the M062x/D95*//M062x/6-31+G** level of theory in the aqueous phase, Table S3: The nuclear relaxation energy, energy barrier G, activation energy *E*_a_, hole transfer rate constant *K*_HT_, and electron coupling energies *V_da_* calculated at the M062X/D95*//M062x/6-31+G** level of theory in the aqueous phase, Table S4: The Ground and Excitation state energies and Excitation and HOMO energies as well as corresponding Dipole Moments (Ground Excitation and Transition) of base pair dimers extracted from *ds*-oligonucleotides, calculated at the M062x/D95*//M062x/6-31+G** level of theory in the aqueous phase using the DFT or TD-DFT methodology, Table S5 Hirshfeld charge and spin distribution in the shape of *ds*-oligonucleotides ^oxo^G_2,4_DNA, RcdA^oxo^G_2,4_DNA, ScdA^oxo^G_2,4_DNA, only nucleosides bases were taken into consideration, calculated at the M062X/D95*//M062x/6-31+G** level of theory in the aqueous phase, Table S6: Base pair Adiabatic (AIP) and Vertical (VIP) Ionisation Potential extracted from *ds*-oligonucleotides: ^oxo^G_2,4_DNA, RcdA^oxo^G_2,4_DNA, ScdA^oxo^G_2,4_DNA, calculated at the M062X/D95*//M062x/6-31+G** level of theory in the aqueous phase, Table S7: Energies (in Hartree) of Neutral, Vertical Cation, Adiabatic Cation and Vertical Neutral forms of *ds*-trimers extracted from *ds*-oligonucleotides calculated at the M062x/D95*//M062x/6-31+G** level of theory in the aqueous phase, Table S8: Root-Mean-Square Deviation of atomic positions (RMSD) in Å, calculated for *ds*-oligonucleotides in their Neutral and Cationic forms, Table S9: Aromatic ring overlaps (in Å^2^) of base pair dimers extracted from ds-oligonucleotides. Geometries, optimized at the M062x/D95* level of theory in the aqueous phase, Table S10: Stacking Energy *E*_ST_ of base pair dimers extracted from *ds*-oligonucleotides, calculated at the M062x/D95*//M062x/6-31+G** level of theory in the aqueous phase using.
